# Supplementary material for: Urbanization alters small rodent community composition but not abundance
Source: PeerJ. 2018 May 30;6:e4885. doi: 10.7717/peerj.4885 (PMC5984581; doi:10.7717/peerj.4885)

**Table S1**

Abundance (# individuals / 100 trap nights) of each species identified in each of the sites surveyed, organized by genus. The urban sites used in this study consist of Piestewa Peak Park (PWP), Desert Botanical Garden (DBG), and South Mountain Park East (SME) and West (SMW). The rural sites are Usery Mountain Regional Park (UMP), Lost Dutchman State Park (LDP), Salt River Recreation (SRR), and north McDowell Mountain Regional Park (MCN).


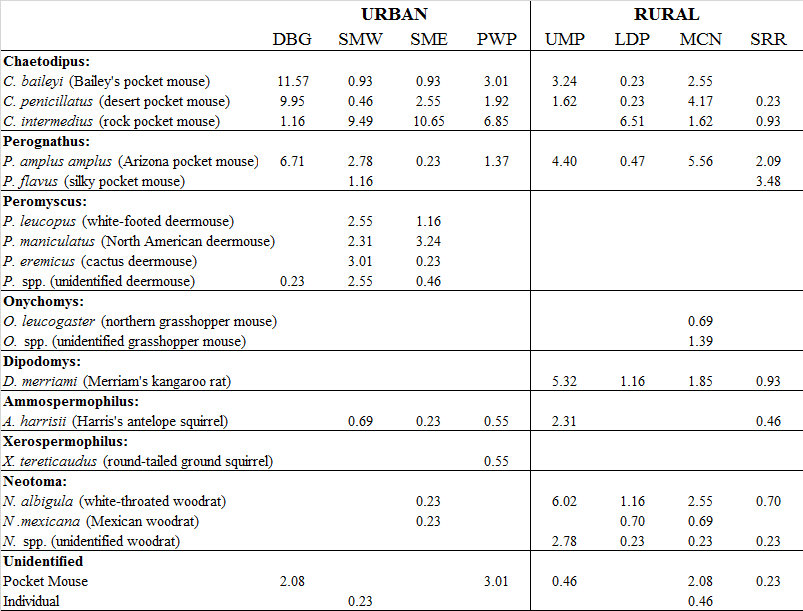

Supplement: Table S1 — The urban sites used in this study consist of Piestewa Peak Park (PWP), Desert Botanical Garden (DBG), and South Mountain Park East (SME) and West (SMW). The rural sites are Usery Mountain Regional Park (UMP), Lost Dutchman State Park (LDP), Salt River Recreation (SRR), and north McDowell Mountain Regional Park (MCN). [file peerj-06-4885-s001.docx]
